# Novel drug discovery platform for spinocerebellar ataxia, using fluorescence technology targeting β-III-spectrin

**DOI:** 10.1074/jbc.RA120.015417

**Published:** 2020-12-24

**Authors:** Robyn T. Rebbeck, Anna K. Andrick, Sarah A. Denha, Bengt Svensson, Piyali Guhathakurta, David D. Thomas, Thomas S. Hays, Adam W. Avery

**Affiliations:** 1 Department of Biochemistry, Molecular Biology and Biophysics, University of Minnesota, Minneapolis, Minnesota, USA; 2 Department of Chemistry, Oakland University, Rochester, Michigan, USA; 3 Department of Genetics, Cellular Biology, and Development, University of Minnesota, Minneapolis, Minnesota, USA

**Keywords:** drug screening, actin binding, fluorescence, time-resolved FRET, fluorescence lifetime, HEK293-6E cells, swinholide A, ABD, actin-binding domain, CH, calponin homology, DMSO, dimethyl sulfoxide, F-actin, actin filaments, FLT, fluorescent lifetime, FRET, fluorescence resonance energy transfer, HEK293-6E, human embryonic kidney suspension cells expressing the Epstein Barr virus nuclear antigen 1, HTS, high-throughput screening, LOPAC, library of pharmacologically active compounds, NPPB, 5-Nitro-2-(3-phenylpropylamino)benzoic acid, PR, plate reader, SCA5, spinocerebellar ataxia type 5

## Abstract

Numerous diseases are linked to mutations in the actin-binding domains (ABDs) of conserved cytoskeletal proteins, including β-III-spectrin, α-actinin, filamin, and dystrophin. A β-III-spectrin ABD mutation (L253P) linked to spinocerebellar ataxia type 5 (SCA5) causes a dramatic increase in actin binding. Reducing actin binding of L253P is thus a potential therapeutic approach for SCA5 pathogenesis. Here, we validate a high-throughput screening (HTS) assay to discover potential disrupters of the interaction between the mutant β-III-spectrin ABD and actin in live cells. This assay monitors FRET between fluorescent proteins fused to the mutant ABD and the actin-binding peptide Lifeact, in HEK293-6E cells. Using a specific and high-affinity actin-binding tool compound, swinholide A, we demonstrate HTS compatibility with an excellent Z’-factor of 0.67 ± 0.03. Screening a library of 1280 pharmacologically active compounds in 1536-well plates to determine assay robustness, we demonstrate high reproducibility across plates and across days. We identified nine Hits that reduced FRET between Lifeact and ABD. Four of those Hits were found to reduce Lifeact cosedimentation with actin, thus establishing the potential of our assay for detection of actin-binding modulators. Concurrent to our primary FRET assay, we also developed a high-throughput compatible counter screen to remove undesirable FRET Hits. Using the FRET Hits, we show that our counter screen is sensitive to undesirable compounds that cause cell toxicity or ABD aggregation. Overall, our FRET-based HTS platform sets the stage to screen large compound libraries for modulators of β-III-spectrin, or disease-linked spectrin-related proteins, for therapeutic development.

β-III-spectrin is one of several conserved cytoskeletal proteins, including α-actinin, filamin, and dystrophin, with mutations in its N-terminal actin-binding domain (ABD) that are linked with human diseases (1, 2, 3, 4, 5, 6, 7, 8). β-III-spectrin forms a heterotetrameric complex with α-II-spectrin and cross-links actin filaments to form a cytoskeleton localized to the shafts and spines of Purkinje cell dendrites (9). We recently determined the molecular consequence of an autosomal dominant mutation (L253P) in the ABD of β-III-spectrin that is linked to spinocerebellar ataxia type 5 (SCA5), a progressive neurodegenerative disease (10). SCA5 is characterized neurologically by ataxia, dysarthria, and eye movement abnormalities such as gaze-evoked nystagmus (11). A key pathological finding is cerebellar hypoplasia, the loss of Purkinje cells in the cerebellar cortex (11, 12). Intriguingly, our recent work revealed that an SCA5-associated missense mutation, L253P, causes a 1000-fold increase in actin-binding affinity (10). Using *Drosophila*, we demonstrated that the SCA5 L253P mutation reduces the plasticity of the spectrin-actin cytoskeleton underlying the plasma membrane, leading to destabilization and loss of dendritic branches (13). This suggests that the cellular mechanism underlying SCA5 pathogenesis is a Purkinje cell deficit connected to loss of dendritic arborization, resulting from elevated actin-binding affinity of the mutant β-III-spectrin. Thus, an effective therapeutic strategy for SCA5 should seek to ameliorate the aberrant actin affinity. Significantly, missense mutations in the highly conserved ABDs of α-actinin and filamin have also been shown to cause increased actin-binding affinity (2, 3, 4, 5, 6, 7). Consequently, small molecules developed for SCA5 therapy may also be useful in treatment of diseases arising from ABD mutations in α-actinin and filamin.

The β-III-spectrin ABD is comprised of tandem calponin homology (CH) subdomains (CH1 and CH2) that are closely associated with one another in a compact structural state. Significantly, the leucine residue that is mutated in SCA5 is positioned at the interface between CH1 and CH2, providing hydrophobic contacts that stabilize the close apposition of the CH subdomains. Biophysical measurements indicated that when the L253P mutation is introduced, the ABD remains well-folded but becomes significantly destabilized (10). This suggested that high-affinity actin binding results from an “opening” of the ABD by disrupting the CH1/CH2 interface. L253P-induced structural opening of the ABD was confirmed by double electron–electron resonance spectroscopy (14). Thus, we propose that small-molecule drugs targeting the mutant ABD can reduce actin binding by either: 1) promoting a shift from the “open” to “closed” conformation, or 2) masking residues in CH1 that are exposed in the “open” state and directly mediate interaction with actin. Moreover, because the mutant ABD populates the “open” conformation more significantly than wild-type, small molecules recognizing the “open” state should selectively target the mutant ABD over wild-type. The similar position of α-actinin and filamin disease-associated mutations at the CH1/CH2 interface predicts that the SCA5 structural mechanism of disease is conserved across the spectrin family of cytoskeletal proteins.

Currently there is no HTS assay that can measure the binding activity of mutant ABDs to actin filaments (F-actin) and can be implemented for drug discovery. Moreover, we are unaware of any effort to develop therapies for diseases resulting from dominant (gain-of-function) mutations in the spectrin family of cytoskeletal proteins. The binding of ABD proteins to actin filaments can be quantified *in vitro* by F-actin cosedimentation assays using purified actin and ABD proteins (10). However, these *in vitro* assays cannot be performed with the throughput required for primary screening of small molecules and instead are most appropriate for validating compound mode of action. Thus, there is an urgent need for an HTS assay that can report on the binding of mutant ABD proteins to actin.

Here we report our development of a fluorescence resonance energy transfer (FRET) assay that detects the binding of the mutant β-III-spectrin L253P ABD to F-actin in cultured mammalian cells. We further demonstrate the feasibility of this assay for HTS using the 1280-compound library of pharmacologically active compounds (LOPAC) in 1536-well plates.

## Results

### Biosensor development and tool compound characterization

**Figure 1:**
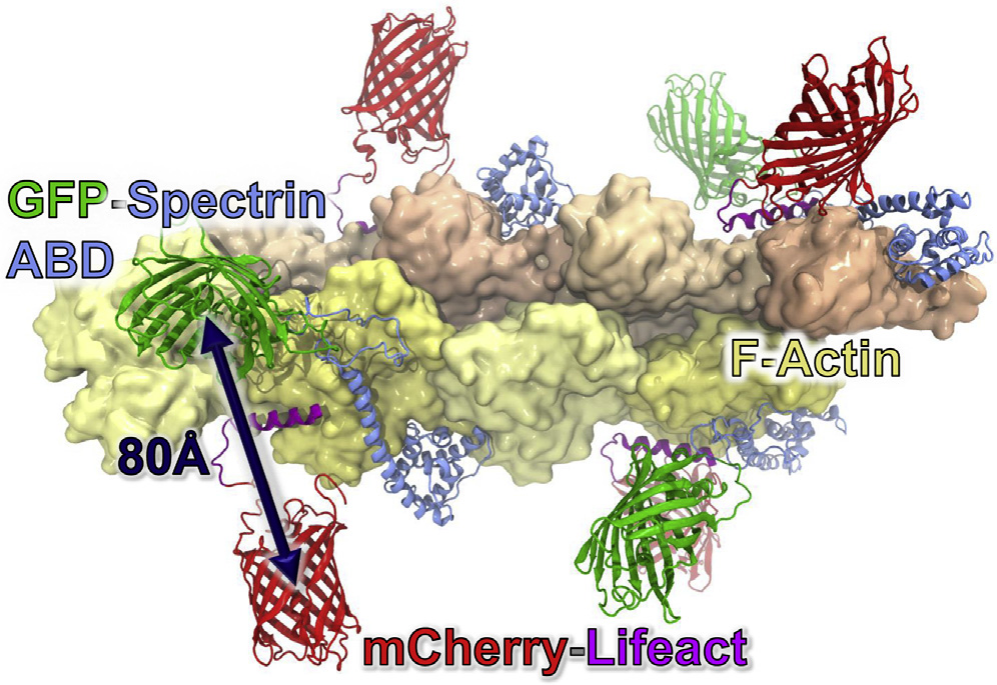
**Model of ABD biosensor.** Model of GFP-ABD-L253P and Lifeact-mCherry bound to F-actin. Position of β-III-spectrin ABD L253P and Lifeact on actin was based on the cryo-EM structures 6ANU (14) and 7TBE (48) and modeled using DS Visualizer (Dassault Systemes, San Diego, CA, USA). As the binding sites for Lifeact and spectrin on actin overlap, every other actin molecule is populated with Lifeact-mCherry or GFP-β-III-spectrin ABD. Molecular figure was generated using VMD (49).

To develop a biosensor that monitors the binding of mutant L253P β-III-spectrin to actin filaments in live cells, a FRET approach was tested. A FRET donor construct consisting of green fluorescent protein fused to the N terminus of the mutant ABD (GFP-ABD-L253P) was coexpressed in HEK293-EBNA1-6E (HEK293-6E) cells with a FRET acceptor construct consisting of the actin-binding peptide, Lifeact, fused to the N terminus of red fluorescent protein, mCherry (Lifeact-mCherry). Lifeact is a 17 amino acid peptide derived from the yeast actin-binding protein, ABP140 (15). The Lifeact-mCherry fusion protein binds specifically to actin filaments (K_d_ = 13.2 μM) (16), with negligible affinity for actin monomers. This micromolar affinity is substantially lower than the 75 nM affinity of our GFP-ABD-L253P protein for actin (10). The cryo-EM structures of ABD L253P and Lifeact on actin indicate that the binding sites overlap on actin units. With ABD L253P and Lifeact bound on neighboring actin units, we predicted an average distance of ∼80 Å between GFP and mCherry (Fig. 1). With a known Förster distance R_0_ of 52.4 Å (17), this corresponds to FRET value of 0.07, a sufficient signal for a biosensor platform. To design a system most compatible for large production and eventual screening of 50,000+ compound libraries, we used HEK293-6E cells, a suspension cell line that yields greater levels (>threefold) of recombinant protein expression (18, 19).

**Figure 2:**
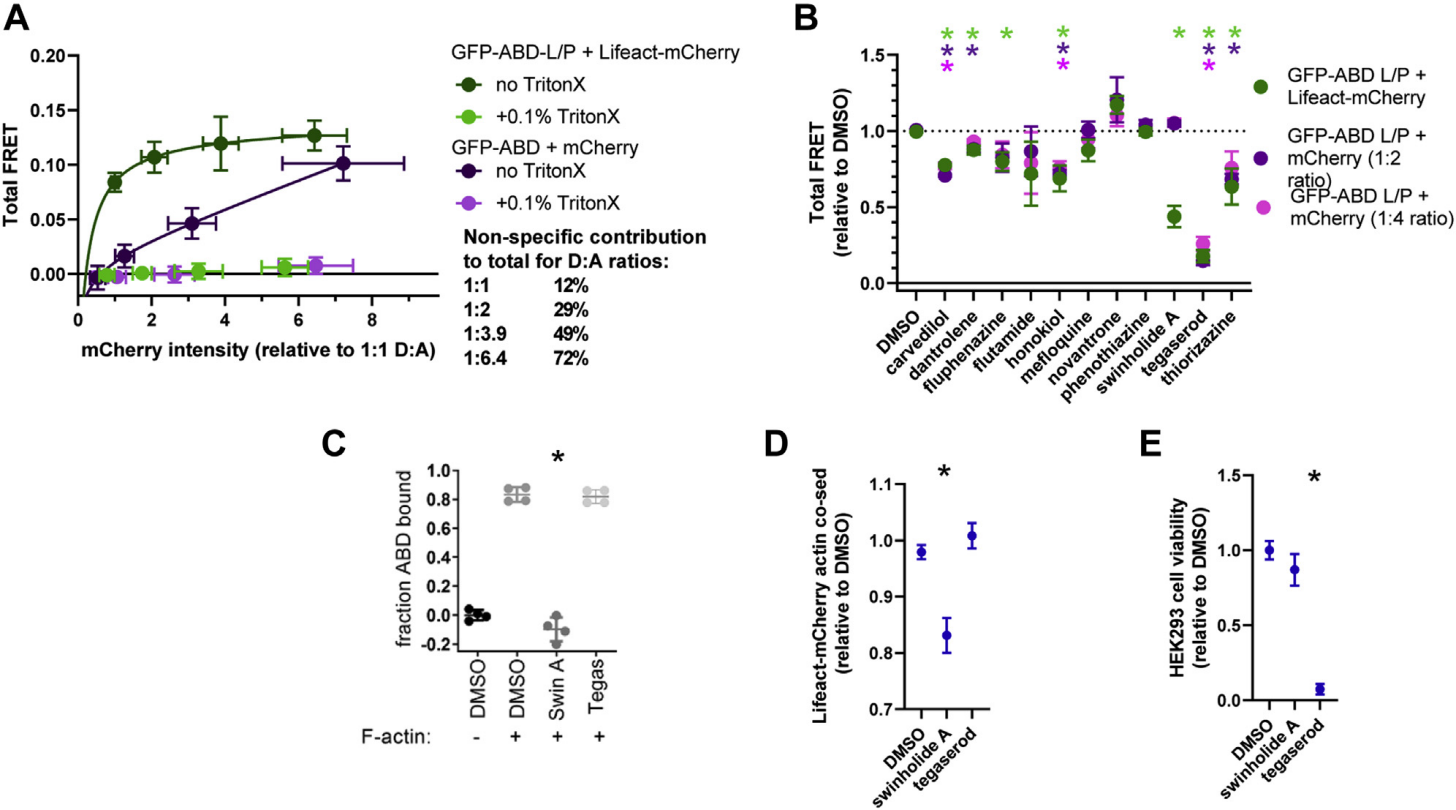
**Measuring specific FRET and identifying potential tool compound, swinholide A, using suspension cells.***A*, total (GFP-ABD and Lifeact-mCherry) and nonspecific (GFP-ABD and mCherry) FRET with shifting donor (GFP) to acceptor (mCherry) ratios in HEK293-6E cells. HEK293-6E cells were transfected with 12% GFP-ABD, and 0, 12, 24, and 84% Lifeact-mCherry or mCherry DNA (% of total 20 μg DNA). For quantified sample comparison, mCherry intensity values were normalized to the mCherry intensity of 1:1 GFP-ABD:Lifeact-mCherry. Total and nonspecific FRET is abolished by addition of 0.1% Triton X-100 for cell lysis. The nonspecific FRET readout demonstrates an increase in nonspecific contribution to the total FRET with an increase in Lifeact-mCherry expression (n = 3). *B*, effect of swinholide A (3 μM) and other actin-binding compounds (50 μM) on total FRET (GFP-ABD L253P plus Lifeact-mCherry) and nonspecific FRET (GFP-ABD plus mCherry) in HEK293-6E cells. The chemical structures of these compounds are shown in Fig. S2. For direct comparison of compound effect on primary and counter biosensors, data is shown as relative to DMSO control (0.123 ± 0.008 for GFP-ABD + Lifeact-mCherry 1:2 ratio; 0.059 ± 0.012 for GFP-ABD + mCherry 1:2 ratio; and 0.117 ± 0.002 for GFP-ABD + mCherry 1:4 ratio) as mean±SD, n = 3. *C*, cosedimentation of ABD and actin showing the ablation of ABD-L253P to actin binding by 3 μM swinholide A (Swin A) and no significant effect of 50 μM tegaserod (Tegas) (n = 4). *D*, cosedimentation of Lifeact-mCherry and actin assays show that 3 μM swinholide A, not 50 μM tegaserod, reduces Lifeact-mCherry binding with actin. Data shown as relative to DMSO control as mean ± SD, n=3 to 4. *E*, trypan blue–based cell viability assay shows that 50 μM tegaserod, not 3 μM swinholide A, abolishes HEK293-6E cell viability. Data is shown as relative to DMSO control as mean ± SEM, n= 3 to 5. ∗Significantly different from DMSO control, *p* < 0.05.

With a constant amount of GFP-ABD-L253P expressed, increased Lifeact-mCherry expression increases FRET with an exponential plateau to 0.12 (Fig. 2*A*). To measure the nonspecific component of our FRET readout, we concurrently tested expression of mCherry in place of Lifeact-mCherry. For comparison, the Lifeact-mCherry and mCherry fluorescence intensity values (normalized to 1:1 GFP-ABD:Lifeact-mCherry sample) are plotted on the X-axis for samples transfected with different donor-to-acceptor DNA ratios. The calculated FRET in our control mCherry cells demonstrates an increase in nonspecific FRET with increasing expression of mCherry, although in a linear fashion (Fig. 2*A*). With the trade-off between high FRET and minimal nonspecific FRET, the donor:acceptor ratio 1:2 was chosen for the experiments that followed (Fig. 2*A*). Cell lysis by addition of 0.1% Triton X-100 abolished both total FRET (GFP-ABD-L253P and Lifeact-mCherry co-expression) and nonspecific FRET (GFP-ABD-L253P and mCherry co-expression), suggesting that our FRET readout is responsive to compounds that alter cell viability and not singularly to compounds altering ABD-actin binding. Of note, we also tested the biosensor using WT-ABD, which yielded substantially lower FRET (Fig. S1), consistent with the 1000-fold decrease in affinity of WT-ABD for actin (10). We proceeded to test the compatibility of the GFP-ABD-L253P and Lifeact-mCherry assay for primary compound screening and the GFP-ABD-L253P and mCherry assay as a potential high-throughput counter screen.

To identify a potential positive control tool compound for our FRET assay, we tested the effect of a range of actin-binding compounds (20, 21) dissolved in DMSO on our FRET readouts. The guiding qualities of a good tool compound include 1) altering only specific FRET, 2) altering the output to the level of the possible signal window, and 3) exhibiting a saturable effect. As shown in Figure 2*B*, seven of 11 actin-binding compounds significantly shift our GFP-ABD-L253P and Lifeact-mCherry coexpression (primary) FRET readout. Notably, the presence of 2% DMSO does not significantly alter our FRET readout (Fig. S1). All FRET effects were shown as relative to DMSO, accommodating for the subtle variability in FRET values across days. Notably, both 1 μM swinholide A and 50 μM tegaserod reduced Total FRET by >50% in our primary FRET assay, suggesting that these may be useful tool compounds (Fig. 2*B*). However, tegaserod, as observed for several of the actin-binding compounds, exhibited a similar impact on the counter-screen FRET assay samples (Fig. 2*B*), suggesting that tegaserod reduces the primary FRET readout by an indirect mechanism such as compound fluorescence or cytotoxicity. Indeed, addition of tegaserod did not alter actin binding of ABD (Fig. 2*C*) nor Lifeact (Fig. 2*D*) in cosedimentation assays, but greatly diminished cell viability (Fig. 2*E*) in trypan blue assays. This suggests that the tegaserod-induced 83% FRET reduction is due to cell lysis, given the 92% decrease in cell viability (Fig. 2). In contrast, swinholide A did not significantly alter the counter-screen FRET readout (Fig. 2*B*) nor cell viability (Fig. 2*E*). Thus, the swinholide A mediated reduction in FRET by 56 ± 7% is likely specific (Fig. 2*B*). Furthermore, this large decrease approaches the maximum specific FRET reduction expected (71%, Fig. 2*A*) for total FRET values, suggesting that swinholide A nearly abolishes specific FRET. The impact of swinholide A on specific FRET, by reducing ABD and Lifeact binding to F-actin, is supported by the large shift in ABD and Lifeact-mCherry from cosedimentation sample actin pellets to supernatants (Fig. 2, *C*–*D*). Thus swinholide A is an ideal tool compound to evaluate the primary FRET assay for HTS.

**Figure 3:**
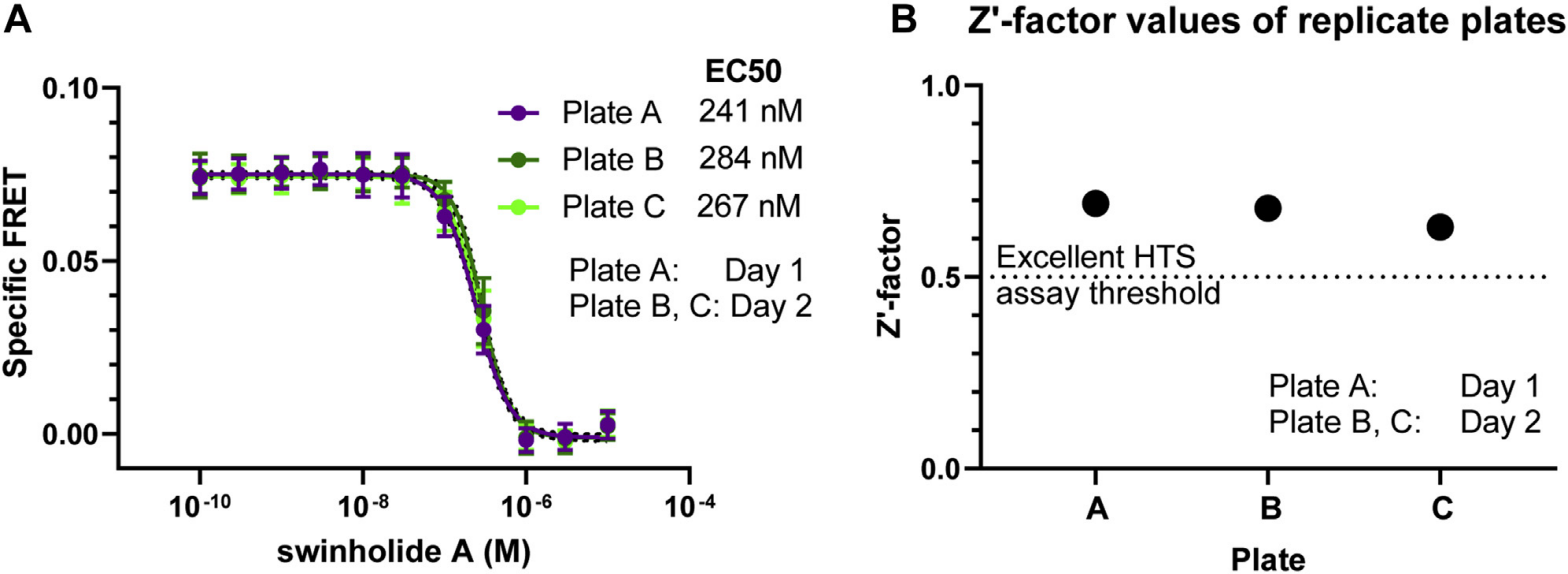
**Swinholide A is a reproducible, positive control tool compound for FRET assays in 1536-well plates.***A*, specific FRET dose response of swinholide A shows reproducible EC50 values across plates read on the same day and different days. For each plate, each concentration was loaded over 64 wells on a 1536-well plate, n = 3 plates. *B*, plot of Z’-factor values for 1 μM swinholide A loaded over 768 wells *versus* DMSO control loaded over 768 wells on a 1536-well plate. Data shown per plate n = 3.

To evaluate the quality of the assay, we used the tool compound, swinholide A, to determine assay robustness and reproducibility. Triplicate, 12-point concentration response curves (0.01 and 30 μM *versus* DMSO-only; 12 points, half log) show that swinholide A reduces FRET in a dose responsive manner, with an average EC50 value of 264 nM (Fig. 3*A*). This is in the range of previously reported affinity values in an actin sedimentation assay (22). To gauge HTS assay robustness, we used swinholide A to measure the Zʹ value, which is a measure of the signal window and data variation between control and tool compound effect. Classically, a value of 0.5 ≤ Z′ < 1 indicates an excellent assay that is ready for large-scale HTS (23). Using 1536-well plates containing half swinholide A and half DMSO negative control, an average Z’-factor of 0.67 ± 0.03 was calculated (Fig. 3*B*), easily exceeding the 0.5 Z’-factor excellence threshold. Significantly, Z’ and EC50 values were highly reproducible when measured in repeat tests performed within the same day, or on different days, using different batches of transfected cells (Fig. 3). These results indicate compatibility of the assay for HTS.

### HTS performance

To test the performance of the assay in HTS, the 1280 LOPAC compounds, together with 256 DMSO controls, were dispensed in 5 nl volumes into individual wells of 1536-well microplates and stored at −20 °C until use. To test the reproducibility of replicate screens on the same day, a single batch of HEK293 cells was transfected with GFP-ABD-L253P and Lifeact-mCherry. In addition, control cells were transfected with only GFP-ABD-L253P, to determine compound effect on donor-only lifetime (τ_D_). To test reproducibility of the screens across different days, identical transfections were performed on another batch of HEK293-6E cells on a second day. Following plate thaw, 5 μl of assay cells were loaded in each well *via* a multidrop liquid dispenser, and a time course of compound effects (at 20, 120, and 180 min post load) on fluorescence lifetime was acquired using a fluorescence lifetime plate reader (FLT-PR). This technology has been advanced to high-density 1536-well plates in recent years for successful HTS using a range of protein biosensors including sarcoplasmic reticulum Ca-ATPase (24, 25, 26, 27), ryanodine receptor (23, 28), actin (20), tumor necrosis factor receptor 1 (29, 30), and tau (31).

**Figure 4:**
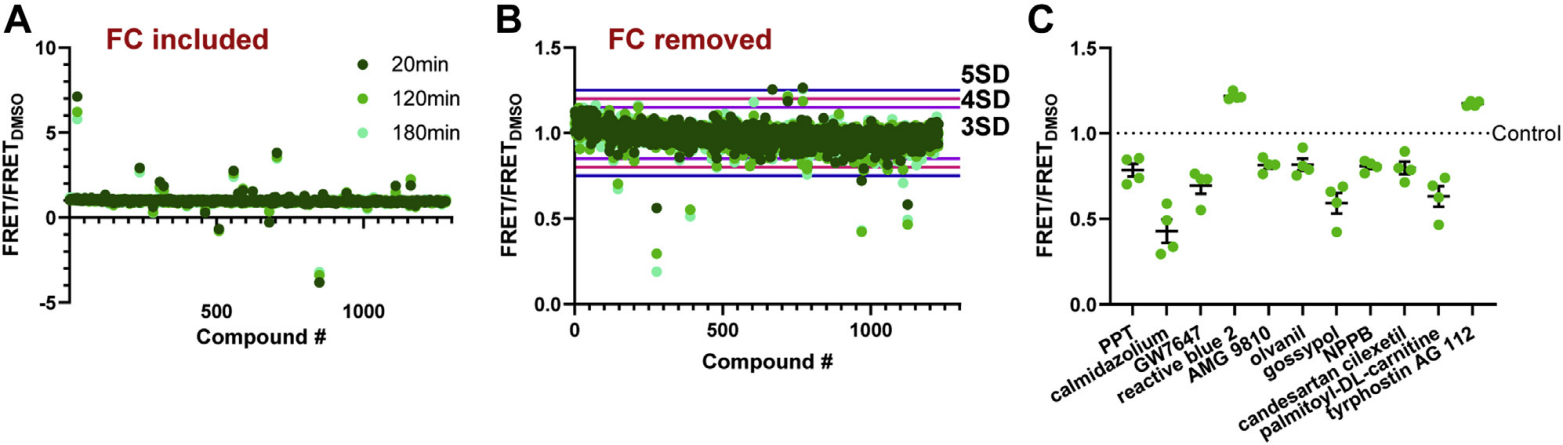
**HTS performance validation of ABD-Lifeact FRET assay using the library of pharmacologically active compounds in 1536-well plates.***A*, FLT data was acquired at three time points following FRET assay loading into 1536-well plates that were preloaded with 1280 library compounds (10 μM final) or DMSO control. Representative FRET response to LOPAC compounds, including interfering, fluorescence compounds (FC). *B*, representative FRET response with interfering compounds removed demonstrates that many Hit compounds have a time dependent effect, with the greatest effect at 120 and 180 min following plate loading. *C*, relative FRET effect of LOPAC Hits that were identified (with 4SD threshold) in at least two of the four screens. n = 4, data shown as mean ± SEM.

Fluorescent interfering compounds were identified as compounds that altered τ_D_ FLT by > 3 standard deviation (SD) threshold and/or altered the fluorescence spectrum by > 3SD, as previously described (23, 24, 25, 28, 32). As shown by representative data in Figure 4, *A*–*B*, several interfering compounds dramatically overshadow the impact of noninterfering Hits. In particular, Figure 4*A* appears to suggest that compound effect on FRET is lowered over time. However, removal of interfering compounds, shown in Figure 4*A*, visibly indicates that Hit compound effects are enhanced over time. Indeed, the overall Hit rates generally increased between 20 and 120 min (Table 1 and Table S1). Given that 120 min incubation yields the highest Hit rate values in all plates, all FRET data from this point onward refers to data acquired 120 min following compound loading.

**Table 1:** Number (#) of Hits and Hit reproducibility for 4 standard deviation (SD) threshold

Description	LOPAC day 1 plate 1	LOPAC day 1 plate 2	LOPAC day 2 plate 1	LOPAC day 2 plate 2
# of Hits 20 min (hit rate%)	18 (1.4%)	23 (1.8%)	28 (2.2%)	26 (2%)
# of Hits 120 min (hit rate%)	26 (2%)	34 (2.7%)	41 (3.2%)	32 (2.5%)
# of Hits 180 min (hit rate%)	26 (2%)	26 (2%)	33 (2.6%)	25 (2%)
% of Repeated Hits in 2 plates^a^	88.5%	79.4%	75.6%	84.4%
% of Repeated Hits in 3 plates^a^	84.6%	64.7%	56.1%	68.8%
% of Repeated Hits in 4 plates^a^	80.8%	61.8%	51.2%	62.5%

a Data for 120 min incubation.

For an acceptable Hit rate (0.5%–3%) (33), we adopted a 4 SD threshold for Hits, which yielded a Hit rate range of 2 to 3.2% (Table 1), compared with the 3.8 to 5.4% and 1.6 to 2% for 3 SD and 5 SD thresholds, respectively (Table S1). At the 4 SD threshold, compounds that were identified as Hits in one screen typically repeated as Hits in replicate screens. As shown in Table 1, the reproducibility of Hit compounds in more than 1, 2, and 3 screens is 82.0 ± 2.8%, 68.6 ± 6.0%, and 64.1 ± 6.1%, respectively. Furthermore, there is little variability in the magnitude of FRET change induced by compounds that were identified in at least two of the four screens (Fig. 4*C* and Fig. S3). Of the 11 Hits, two increased FRET and nine decreased FRET (Fig. 4*C*). Notably, none of these Hit compounds are known to bind to actin, Lifeact, nor the β-III-spectrin ABD. Because our ultimate interest is to identify compounds that reduce the aberrant affinity of mutant β-III-spectrin for actin, we further characterized the nine hits that reduced FRET.

### FRET dose–response assay

**Figure 5:**
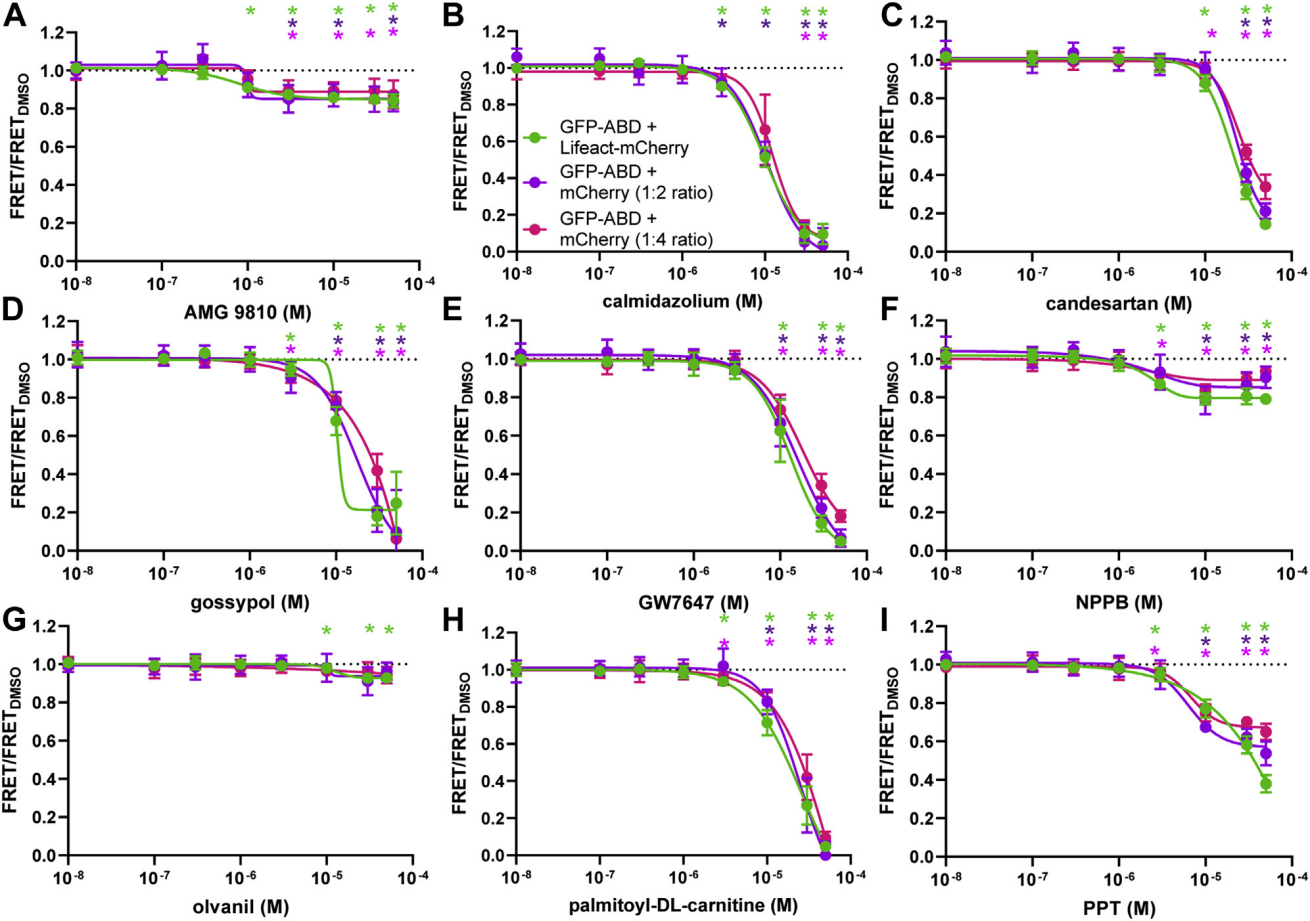
**FRET dose response of Hit compounds that decrease FRET.***A*–*I*, dose response of Hit compounds AMG 9810 (*A*), calmidazolium (*B*), candesartan (*C*), gossypol (*D*), GW7647 (*E*), NPPB (*F*), olvanil (*G*), palmitoyl-DL-carnitine (*H*), and PPT (*I*) were tested on the primary screen (GFP-ABD-L253P to Lifeact-mCherry FRET; 1:2 ratio) and two counter screens (GFP-ABD-L253P to mCherry nonspecific FRET; 1:2 and 1:4 ratios). The chemical structures of these Hit compounds are shown in Fig. S4. Data shown as mean ± SEM, n = 3.

To determine dose–response relationships, we measured the FRET response to a range of Hit compound concentrations under the same assay conditions as used in the primary screen. Further, we also tested how Hit compounds altered FRET in our counter screen (GFP-ABD-L253P and mCherry). All nine repurchased compounds decreased counter FRET to similar levels observed in the primary screen (Figs. 4 and 5). This indicates that our Hit threshold in the primary screen is sufficient to identify FRET effectors. Curiously, all compounds displayed similar effects on counter-screen FRET, suggesting that these compounds alter fluorescence, cell viability, or protein aggregation. Our assay is sensitive to modulators that shift FRET by as little as 2 to 14% at 10 μM, specifically AMG 9810, candesartan, and olvanil (Fig 5).

### Characterization of Hit compound mode of action

**Figure 6:**
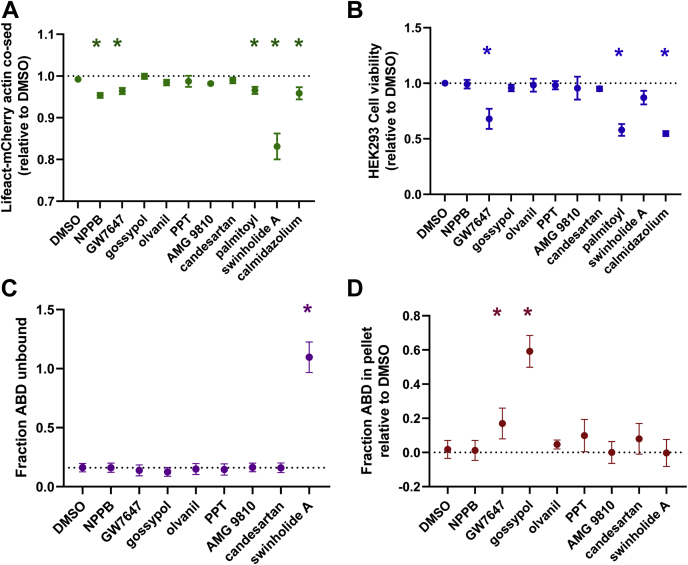
**FRET Hit mode of action evaluated using Lifeact-mCherry binding to actin, cell viability, ABD-L253P binding to actin, and ABD-L253P aggregation.***A*, cosedimentation of Lifeact-mCherry and actin assays show that 3 μM swinholide A, and only 10 μM HTS Hits NPPB, GW7647, palmitoyl-DL-carnitine, and calmidazolium reduces Lifeact-mCherry binding with actin. Data shown as relative to DMSO control as mean±SEM, n=3 to 5. *B*, trypan blue–based cell viability assay shows that 3 h incubation with 10 μM GW7647, palmitoyl-DL-carnitine, and calmidazolium, reduces HEK293-6E cell viability. Data shown as relative to DMSO control as mean ± SEM, n=3 to 5. *C*, cosedimentation of ABD and actin show that only 3 μM swinholide A, not 10 μM Hits, reduce ABD-L253P binding to actin. Data is shown as relative to DMSO control as mean ± SEM, n= 3. *D*, sedimentation of ABD L253P (without actin) assays is used to identify compounds that promote aggregation. Data show that 10 μM GW7647 and gossypol promote aggregation. Data is shown as relative to DMSO control as mean ± SEM, n= 4. ∗Significantly different from DMSO control, *p* < 0.05.

To gain insight into Hit compound mode of action, we investigated the impact of compounds on cosedimentation of ABD and Lifeact with actin, cell viability, and ABD aggregation. Addition of 10 μM calmidazolium, GW7647, NPPB, or palmitoyl-DL-carnitine decreased actin-Lifeact cosedimentation, suggesting that these compounds promote Lifeact dissociation from actin (Fig. 6*A*). However, this does not explain the impact of these compounds on the counter screens. Like tegaserod, screen Hits GW7647, palmitoyl-DL-carnitine, and calmidazolium dramatically reduced cell viability (Fig. 6*B*), which would account for compound effect on both the primary screen and counter-screen assays. Indeed, both calmidazolium and palmitoyl-DL-carnitine have been reported to reduce cell viability in mammalian cells (34, 35). Notably, olvanil, gossypol, and candesartan cilexetil have also been previously found to reduce cell viability, though the impact was minimal (<10%) at the concentration (10 μM) used in the present study (36, 37, 38). With consensus between our findings and the literature on the impact of calmidazolium and pamitoyl-DL-carnitine on cell viability, we did not investigate the impact of these compounds further.

Similar to tegaserod, the remaining Hit compounds did not significantly reduce ABD-L253P to actin cosedimentation (Fig. 6*C*). This further supports the quality of the counter screen, as all compounds that impacted the counter screen did not impact ABD-actin binding (Figs. 2, 5 and 6), with the possible exception of olvanil, which subtly reduced primary FRET with no significant effect on counter FRET (Fig. 5). In addition to a reduction in cell viability, the primary and counter screen may be sensitive to compounds that promote ABD-L253P protein aggregation. Indeed, using the aggregator advisor database and tool (39), we identified gossypol, PPT, and candesartan as known protein aggregators (40, 41, 42) and AMG 9810 as having 71% structural similarity with a known aggregator (40). Our assay measuring ABD-L253P cosedimentation with actin is not sensitive to ABD aggregation, as it is designed to work under conditions where ∼90% ABD-L253P cosediments with actin. Nevertheless, we repeated these measurements in the absence of actin. As shown in Figure 6*D*, the presence of 10 μM GW7647, or gossypol, increases ABD pelleting during centrifugation, presumably due to protein aggregation. Notably, PPT and candesartan also trend toward increasing ABD pelleting, consistent with the small effect of PPT and candesartan on FRET at 10 μM. As expected, 1.6 μM swinholide A treatment did not induce ABD sedimentation (Fig. 6*D*). Overall, our data indicates that the counter screen is sensitive to compounds that are detected in our primary screen due to ABD protein aggregation and/or cytotoxicity.

## Discussion

For HTS, we developed a highly quantitative time-resolved FRET assay that reports on the elevated actin affinity induced by the L253P spinocerebellar ataxia type 5 mutation in β-III-spectrin. This live cell assay is based on the formation of a ternary complex consisting of GFP-ABD-L253P, Lifeact-mCherry, and actin. Treatment of cells with the F-actin severing compound, swinholide A, strongly reduced FRET, thus demonstrating the requirement of F-actin to assemble the ternary complex that brings GFP-ABD and Lifeact-mCherry into close proximity with each other. Following assay optimization in HEK293-6E suspension cells and 1536-well microplates, we showed using a high-throughput fluorescence lifetime plate reader (25, 28, 30) that the assay is robust based on high Z’ scores (>0.5) using 1536-well plates containing half DMSO and half swinholide A and is reproducible based on high repeatability of Hits in replicate screens of the 1280-compound LOPAC library. Moving forward, our goal is to apply the optimized assay and protocols reported here to screen larger libraries with greater diversity in molecular scaffolds, to identify small molecules that directly bind the mutant ABD and reduce its affinity for actin. The challenge will be to identify compounds that modulate the actin-binding affinity of mutant β-III-spectrin into the “Goldilocks” zone—where aberrant binding is sufficiently suppressed to prevent disease progression while allowing adequate binding to support proper cell function. A similar strategy of pulsed-drug dosing has proven effective in treating diseases such as leukemia and melanoma, where the oncogenic factor is known to impinge on multiple biological pathways (43).

In addition to the primary high-throughput FRET assay described above, we also developed a high-throughput counter assay to remove Hits that impact the primary FRET assay through undesired modes of action. The counter assay is based on an intermolecular FRET signal measured in cells expressing GFP-ABD-L253P and mCherry. We showed that this “non-specific” intermolecular FRET signal is sensitive to cell lysis, as observed following Triton X-100 or tegaserod treatment (Fig. 2). The counter assay was further validated by Hits identified in our primary screens of the LOPAC library. Eight of nine Hits that lowered FRET in our primary assay (GFP-ABD-L253P and Lifeact-mCherry) had a similar impact on FRET in the counter assay. Our secondary assays showed that decreased FRET caused by at least five of the Hit compounds probably results from compound-induced cytotoxicity or ABD aggregation. The compounds that displayed more subtle effects on FRET at 10 μM (AMG 9810, candesartan, olvanil, and NBBP) do not significantly alter ABD cosedimentation, cell viability, nor aggregation. This could be due to the reduced sensitivity of these *in vitro* secondary assays. The counter assay should also be sensitive to compounds that impact FRET due to intrinsic fluorescence. Moving forward, we will employ the 1:4 ratio GFP-ABD:mCherry expression ratio in the counter assay due to greater signal window and because this ratio appears to reflect the same compound impacts as the 1:2 ratio with smaller signal window. Because of the sensitivity of the counter assay and the ability to perform it with high throughput, it is possible that a higher hit rate can be accommodated in the primary screen by lowering the Hit threshold from 4 SD to 3 SD. Based on LOPAC screening, this would increase the overall Hit rate from 2–3.2% to 3.8–5.4%. The lower threshold would allow the identification of Hits that have a smaller impact on binding of the mutant ABD to actin and increase the probability of identifying Hit compounds with the desired mode of action.

**Figure 7:**
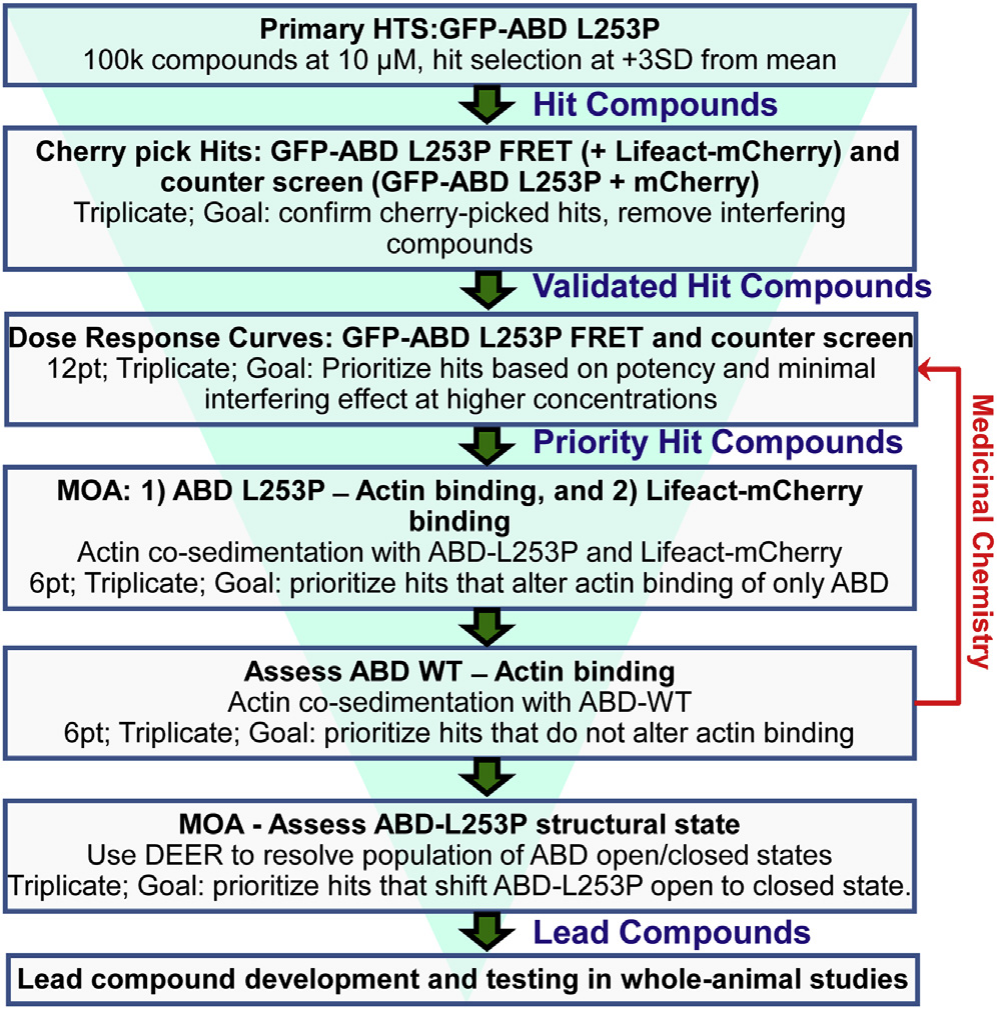
**Proposed ABD L253P HTS platform for drug screening and development starting with a 100,000 compound library screen**.

Our primary and counter screens are designed to yield a pool of Hits enriched with compounds that disrupt the ternary FRET complex and can be further evaluated for their mode of action in secondary assays. Notably, rational design approaches, including pursuit of structural analogues of the tool compound swinholide A and *in silico* docking screening when possible, may identify additional candidate small molecules. As performed here, *in vitro* cosedimentation assays can resolve whether these compounds act directly on the mutant ABD, as desired, or on actin itself. Specifically, we anticipate that compounds that bind to actin will alter Lifeact-mCherry to actin cosedimentation, similar to the impact on ABD-L253P to actin cosedimentation. Transient phosphorescence anisotropy can confirm the impact of compounds on actin structural dynamics (20, 44, 45, 46). Additional cosedimentation assays with the wild-type β-III-spectrin ABD will allow selection of compounds that have greater affinity for the mutant ABD *versus* wild-type. We envision that some compounds that recognize the open conformation of the ABD will preferentially target the mutant ABD over wild-type, because the mutant populates the “open” conformation more significantly than wild-type. A flow chart of our screening strategy is given in Figure 7.

The screening platform and protocols reported here have the potential to identify a molecular scaffold that can be further developed into a therapeutic for SCA5. Such a drug could be used to treat SCA5 patients carrying the L253P mutation and potentially for the other SCA5-linked mutations located at the CH1-CH2 interface that may also increase actin binding (47). Moreover, a drug developed for SCA5 may also be useful in other rare diseases, such as Focal Segmental Glomerulosclerosis, Congenital Macrothrombocytopenia, Otopalatiodigital Syndrome, Autosomal Dominant Atelosteogenesis, William’s Distal Myopathy, which have also been linked to “gain-of-function” ABD mutations in spectrin-related proteins, including filamin (5, 6, 7) and α-actinin (2, 3, 4). Alternatively, we expect that our FRET biosensor could easily be adapted to include the mutant filamin or α-actinin ABD and screening performed as described in Figure 7, to identify compounds specifically targeting the spectrin-related mutant ABDs. Our cell-based assay platform is versatile, and our validated screening protocols provide an inroad to drug discovery for spinocerebellar ataxia and numerous other actin-linked cytoskeletal disorders.

## Experimental procedures

### Compound handling and preparation of 1536-well assay plates

The LOPAC compounds (Sigma-Aldrich, MO, USA) were received in 96-well plates and reformatted into 384-well polypropylene intermediate plates (Greiner Bio-One, Kremsmunster, Austria) using a multichannel liquid handler, BioMek FX (Beckman Coulter, Miami, FL, USA), then transferred to 384-well Echo Qualified source-plates (Labcyte Inc, Sunnyvale, CA, USA). Assay plates were prepared by transferring 5 nl of the 10 mM compound stocks in columns 3 to 22 and 27 to 46 or DMSO in columns 1 to 2, 23 to 26, and 47 to 48 from the source plates to 1536-well black polypropylene plates (Greiner), using an Echo 550 acoustic dispenser (Labcyte Inc). These assay plates were then heat-sealed using a PlateLoc Thermal Microplate Sealer (Agilent Tech., Santa Clara, CA, USA) and stored at −20 °C prior to usage. Swinholide A was purchased from Cayman Chemical (Ann Arbor, MI, USA).

### Molecular biology

The GFP-ABD DNA was constructed with GFP sequence genetically fused to N terminus of the β-III-spectrin (human) ABD residues 1 to 284. The Lifeact-mCherry DNA was a gift from Michael Davidson (Addgene plasmid #54491) and contains mCherry genetically fused to the C terminus of Lifeact. For expression in HEK293-6E cells (National Research Council Canada; Ottawa, Canada), DNA sequences were subcloned into pTT5 vector (National Research Council Canada; Ottawa, Canada) using NheI-HF and NgoMIV restriction enzyme sites. For expression in *E. coli*, the L253P β-III-spectrin ABD and Lifeact-mCherry DNA sequences were subcloned into the BsaI site in the vector pE-SUMOpro (LifeSensors), using AarI and XbaI.

### Cell culture

At 2 × 10^6^ cells/ml, HEK293-6E cells were transfected with GFP-ABD-L253P or GFP-ABD-WT (2.4 μg), Lifeact-mCherry (2.4–16.8 μg), mCherry (1.2–16.8 μg), and/or pTT5 (0.8–17.6 μg), as indicated, using the 293fectin protocol (Thermo Fisher Scientific) with 20 μg total DNA. Cells were transfected with either GFP-ABD-L253P or –WT, but coexpression of the WT and mutant isoform was not explored in this study. The cells were harvested 24 h later by centrifugation at 100*g* for 5 min and then washed twice with PBS. Cell viability and concentration were determined using trypan blue assay and a Countess cell counter (Invitrogen).

### HTS cell preparation and FRET measurements

Using a Multidrop Combi liquid dispenser (Thermo Fisher Scientific), 10^6^ cells/ml were dispersed into 1536-well and 384-well plates as 5 or 50 μl aliquots, respectively. For control and tool compound plates, 100 nl of DMSO or potential tool compound was loaded using the Mantis (Formulatrix). For follow-up retesting of purchased LOPAC screen hits, 384-well plates were loaded with 1 μl compound using a Mosquito LV (SPTLabTech, United Kingdom). Two to three hours after sample loading, fluorescence lifetime measurements were performed using high-throughput fluorescence plate readers provided by Photonic Pharma LLC (MN, USA), including one detecting fluorescence lifetime and another detecting fluorescence spectra, as described previously (20, 24, 25, 32).

### HTS data analysis

Time-resolved fluorescence waveforms for each well were fitted based on a one-exponential decay function using least-squares minimization global-analysis software, as detailed previously (32). The FRET efficiency (*E*) was determined as the fractional decrease of donor fluorescence lifetime (τ_D_), due to the presence of acceptor fluorophore (τ_DA_), using the following equation:

(1) $$E=1-\frac{\tau_{\mathrm{DA}}}{\tau_{D}}$$

Assay quality was determined based on FRET assay samples in wells preloaded with control (DMSO) and tested tool compound, as indexed by the Z΄ factor:

(2) $$Z\prime =1-3\frac{\sigma_{\mathrm{DMSO}}+\sigma_{\mathrm{Tool}}}{\left|\mu_{\mathrm{DMSO}}-\mu_{\mathrm{Tool}}\right|}$$

where σ_DMSO_ and σ_Tool_ are the SDs of the DMSO τ_DA_ and tool compound τ_DA_, respectively; μ_DMSO_ and μ_Tool_ are the means of the DMSO τ_DA_ and tool compound τ_DA_, respectively. A compound was considered a Hit if it changed τ_DA_ by >4SD relative to that of control τ_DA_ that were exposed to 0.1% DMSO.

### Protein preparation

Actin was purified from acetone powder derived from the psoas muscle of New Zealand white rabbit (*Oryctolagus cuniculus*) (46). Filamentous (F)-actin was stored for up to 3 days on ice before use in binding assays. Within an hour of the binding assay, the F-actin was clarified at 100,000*g* at 4 °C for 10 min prior to setting up binding assays. The L253P β-III-spectrin ABD was expressed in *E. coli* BL21(DE3) (Novagen). The ABD was purified and the His-SUMO tag proteolytically removed, as previously described (14), except size-exclusion chromatography was not performed due to the high purity of the ABD protein. Lifeact-mCherry protein was similarly prepared. A Bradford assay was performed to determine clarified F-actin and ABD protein concentrations.

### F-actin cosedimentation assays

F-actin and ABD binding assays were performed as previously described (10). In brief, binding assays used 1 μM ABD protein, 1 μM F-actin, and 1 to 50 μM compound (as indicated) or DMSO control for a total reaction volume of 60 μl in F-buffer containing 10 mM Tris, pH 7.5, 150 mM NaCl, 0.5 mM ATP, 2 mM MgCl_2_, and 1 mM DTT. Following 30 min incubation at room temperature (21 °C). F-actin was pelleted using centrifugation at 100,000*g* at 25 °C for 30 min. Unbound ABD in the supernatant was measured following SDS-PAGE and Coomassie blue staining in accord (10). In addition, control cosedimentations assays that lacked actin were performed to test if compounds cause ABD aggregation. F-actin and Lifeact-mCherry binding assays were undertaken with 2 μM Lifeact-mCherry, 30 μM F-actin, and 1 to 50 μM compound (as indicated) or DMSO control for a total reaction volume of 80 μl in F-buffer. For a no-actin binding control, an assay sample with no F-actin was also set up. Binding reactions were allowed to reach equilibrium at room temperature (21 °C) for 30 min, and then F-actin was pelleted by centrifugation at 100,000*g* at 25 °C for 30 min. The amount of unbound Lifeact-mCherry was sampled by loading 7 μl of spin supernatant on a low-volume black bottom 384-well plate (Greiner). Amount of mCherry was determined by acquiring fluorescence spectrum peak intensity at ∼600 nm using a spectral unmixing plate reader (Fluorescence Innovations, Inc) equipped with a 532 nm laser (Laserglow Technologies, Ontario, Canada) for excitation, 532 nm long-pass filter for emission, and PMT for emission detection. The fraction of Lifeact-mCherry bound to F-actin was determined by subtracting from one the fraction of supernatant fluorescence intensity relative to the no-actin control sample:

(3) $$\mathrm{Lifeact}\;\mathrm{bound}\;\mathrm{fraction}=1-\frac{F_{\mathrm{compound}/\mathrm{DMSO}}}{F_{\mathrm{No}-\mathrm{actin}}}$$

where F_control/DMSO_ is the peak mCherry fluorescence of assay samples containing compound or DMSO control, and F_no-actin_ is the peak mCherry fluorescence of assay samples that contained no F-actin.

### Trypan blue cell viability assays

HEK293-6E cell viability was measured using trypan blue (0.03%) staining and a Countess Automated Cell counter (Invitrogen). Nontransfected HEK293-6E cells were spun at 100*g* for 5 min at 21 °C and resuspended in 10 ml of PBS. The centrifugation and resuspension were repeated twice more. Final cell concentration was 1 × 10^6^ cells/ml. Cells were incubated with 2% DMSO or 3 to 50 μM compound (as indicated) for 3 h at room temperature (21 °C). After addition of 100 μl of 0.06% trypan blue solution in PBS, 10 μl was loaded on a slide and injected into the cell counter, and cell viability was measured.

### Analysis and presentation of data

Data is presented as mean ± SD or ±SEM, as indicated. For statistical difference determination, unpaired Student’s *t*-test was performed. Statistical analyses were performed with GraphPad Prism and Origin. Significance was accepted at *p* < 0.05. EC50 values were derived from fits to Hill equations.

## Data availability

All data are contained within the article.

## Conflict of interest

D. D. T. holds equity in and serves as an executive officer for Photonic Pharma LLC. These relationships have been reviewed and managed by the University of Minnesota. Photonic Pharma had no role in this study, except to provide some instrumentation, as stated in Experimental Procedures. R. T. R., A. K. A., S. A. D., B. S., P. G., T. S. H., and A. W. A. have no conflict of interest to disclose.
