# Supplementary material for: Novel drug discovery platform for spinocerebellar ataxia, using fluorescence technology targeting β-III-spectrin
Source: J Biol Chem. 2020 Dec 24;296:100215. doi: 10.1074/jbc.RA120.015417 (PMC7948455; doi:10.1074/jbc.RA120.015417)
Supplement: Supplementary Figures and Tables [file mmc1.pdf]

Supporting Information

Novel drug discovery platform for spinocerebellar ataxia, using fluorescence technology targeting β-III-spectrin

Robyn T. Rebbeck[1], Anna K. Andrick[1], Sarah A. Denha[2], Bengt Svensson[1], Piyali Guhathakurta[1], David D. Thomas[1], Thomas S. Hays[3] and Adam W. Avery[2,3*]

[1] Department of Biochemistry, Molecular Biology and Biophysics, University of Minnesota, Minneapolis, MN 55455, USA;
[2] Department of Chemistry, Oakland University, Rochester, MI 48309-4479, USA;
[3] Department of Genetics, Cellular Biology, and Development, University of Minnesota, Minneapolis, MN 55455, USA;

***** Corresponding Author: Adam W. Avery
Email: awavery@oakland.edu

Running title: **Early phase drug discovery targeting β-III-spectrin**

Table S1. Number (#) of Hits and Hit reproducibility for 3 and 5 standard deviation (SD) thresholds.

		**LOPAC Day 1 Plate 1**	**LOPAC Day 1 Plate 2**	**LOPAC Day 2 Plate 1**	**LOPAC Day 2 Plate 2**
3SD	# of Hits 20 min (hit rate%)	28 (2.2%)	38 (3%)	40 (3.1%)	41 (3.2%)
	# of Hits 120 min (hit rate%)	49 (3.8%)	56 (4.4%)	69 (5.4%)	64 (5%)
	# of Hits 180 min (hit rate%)	49 (3.8%)	44 (3.4%)	63 (4.9%)	50 (3.9%)
	% of Repeated Hits in 2 plates[1]	77.6%	73.2%	63.8%	62.5%
	% of Repeated Hits in 3 plates[1]	71.4%	66.1%	55.1%	54.7%
	% of Repeated Hits in 4 plates[1]	57.1%	50%	40.6%	43.8%
5SD	# of Hits 20 min (hit rate%)	15 (1.2%)	17 (1.3%)	27 (2.1%)	21 (1.6%)
	# of Hits 120 min (hit rate%)	21 (1.6%)	22 (1.7%)	26 (2%)	25 (2%)
	# of Hits 180 min (hit rate%)	18 (1.4%)	19 (1.5%)	27 (2.1%)	23 (1.8%)
	% of Repeated Hits in 2 plates[1]	90.5%	95.5%	88.5%	88%
	% of Repeated Hits in 3 plates[1]	90.5%	90.9%	76.9%	80%
	% of Repeated Hits in 4 plates[1]	90.5%	86.4%	73.1%	76%

[1] Data for 120 min incubation.

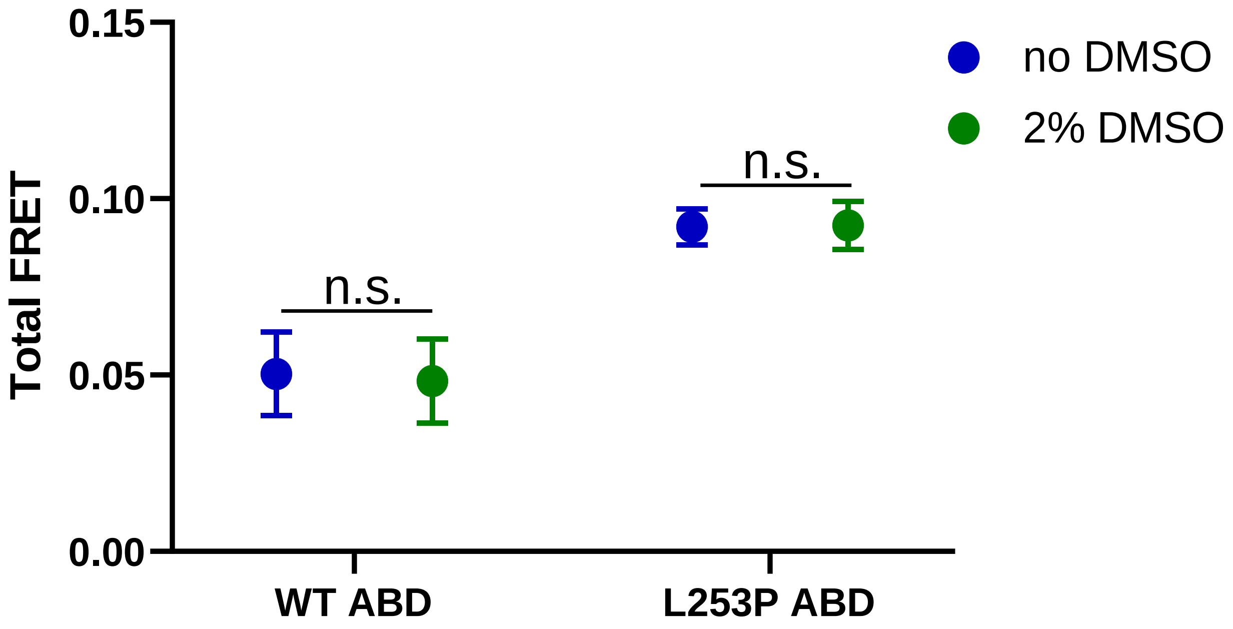

Figure S1. Measuring FRET using GFP-ABD-WT vs. GFP-ABD-L253P with and without DMSO. Total FRET was acquired from HEK293-6E cells expressing recombinant Lifeact-mCherry and GFP-ABD-WT or GFP-ABD-L253P. Incubation with and without 2%DMSO did not significantly alter (n.s.) FRET for each biosensor. Data is shown as relative to DMSO control as mean ± SD, n= 3.

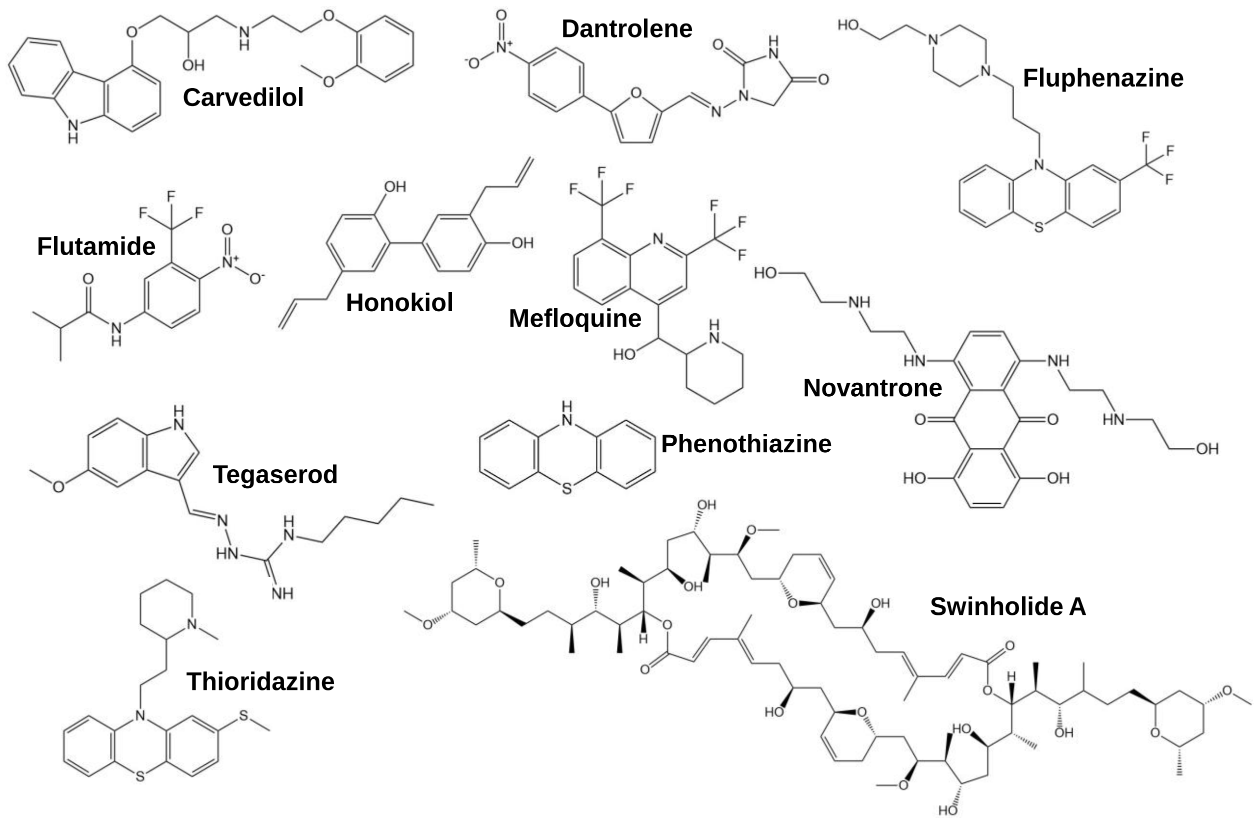

Figure S2. Chemical structures of known actin binding compounds.

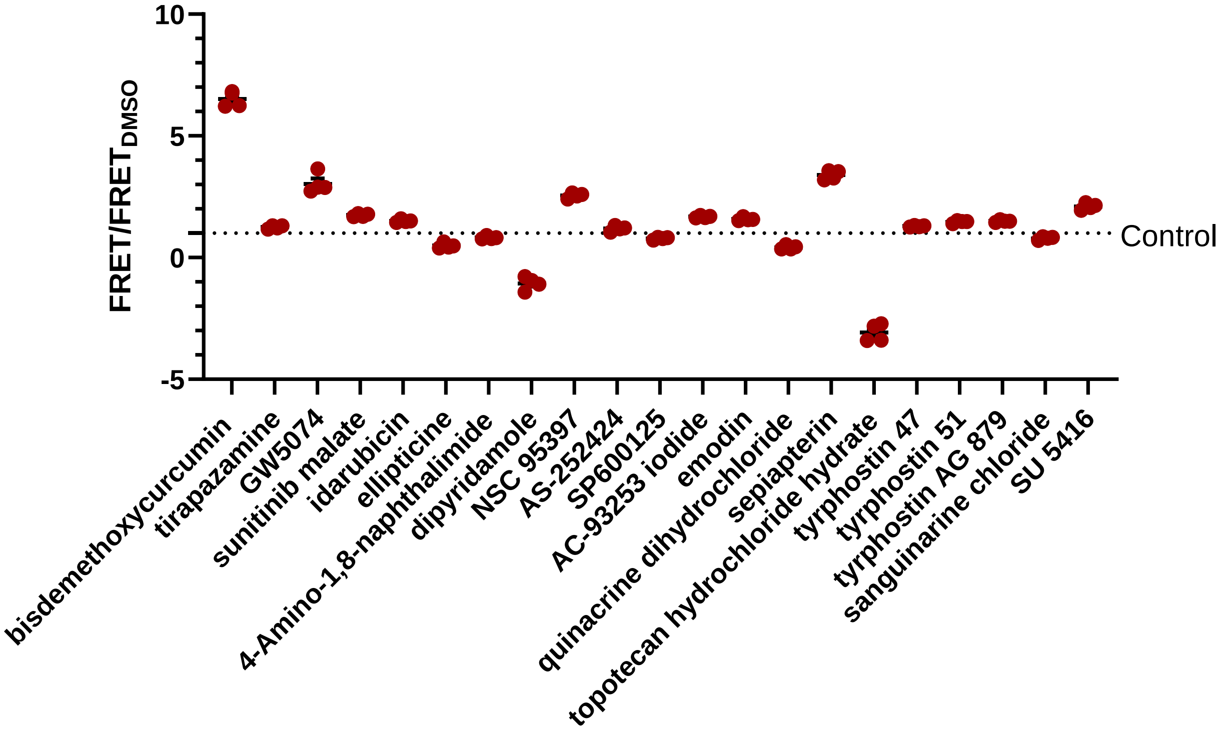

Figure S3. Interfering compounds identified as Hits by ABD-Lifeact FRET assay using the library of pharmacologically active compounds in 1536-well plates. Relative FRET effect of LOPAC Hits that were identified (with 4SD threshold) in at least 2 of the 4 screens, and confirmed as fluorescently interfering compounds by spectral readout, as previously described (23-25,28,32). n = 4, data shown as mean ± SEM.

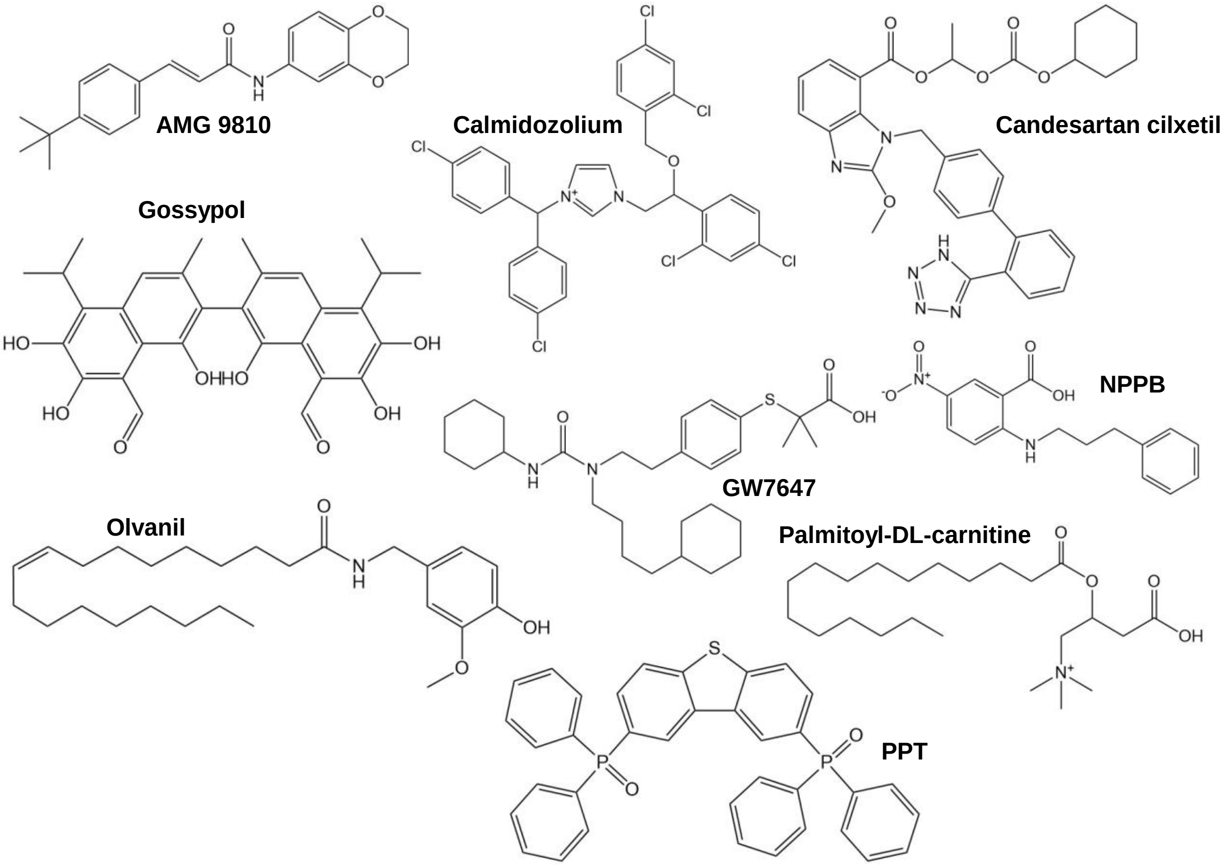

Figure S4. Chemical structures of validated HTS hits tested in dose-response and secondary assays.
